# Ion Concentration- and Voltage-Dependent Push and Pull Mechanisms of Potassium Channel Ion Conduction

**DOI:** 10.1371/journal.pone.0150716

**Published:** 2016-03-07

**Authors:** Kota Kasahara, Matsuyuki Shirota, Kengo Kinoshita

**Affiliations:** 1 Graduate School of Information Sciences, Tohoku University, 6-3-09, Aramaki-aza Aoba, Aoba-ku, Sendai, Miyagi 980–8597, Japan; 2 Tohoku Medical Megabank Organization, Tohoku University, 2–1, Seiryo-cho, Aoba-ku, Sendai, Miyagi 980–8573, Japan; 3 Graduate School of Medicine, Tohoku University, 2–1, Seiryo-cho, Aoba-ku, Sendai, Miyagi 980–8575, Japan; 4 Institute of Development, Aging, and Cancer, Tohoku University, 4–1, Seiryo-cho, Aoba-ku, Sendai, Miyagi 980–8575, Japan; Universidad Autonoma de San Luis Potosi, MEXICO

## Abstract

The mechanism of ion conduction by potassium channels is one of the central issues in physiology. In particular, it is still unclear how the ion concentration and the membrane voltage drive ion conduction. We have investigated the dynamics of the ion conduction processes in the Kv1.2 pore domain, by molecular dynamics (MD) simulations with several different voltages and ion concentrations. By focusing on the detailed ion movements through the pore including selectivity filter (SF) and cavity, we found two major conduction mechanisms, called the III-IV-III and III-II-III mechanisms, and the balance between the ion concentration and the voltage determines the mechanism preference. In the III-IV-III mechanism, the outermost ion in the pore is pushed out by a new ion coming from the intracellular fluid, and four-ion states were transiently observed. In the III-II-III mechanism, the outermost ion is pulled out first, without pushing by incoming ions. Increases in the ion concentration and voltage accelerated ion conductions, but their mechanisms were different. The increase in the ion concentrations facilitated the III-IV-III conductions, while the higher voltages increased the III-II-III conductions, indicating that the pore domain of potassium channels permeates ions by using two different driving forces: a push by intracellular ions and a pull by voltage.

## Introduction

Ion channels are an essential class of proteins that function to transport ions across a plasma membrane [1]. Potassium channels, in particular, play a central role in several high-order physiological phenomena, such as establishing action potentials in heart muscles [2]. They passively transport K^+^ ions with high selectivity from other monovalent cations [3–5]. One of the important characteristics of potassium channels is their precise control of ion conductance. Although many kinds of potassium channels exist with unique gating mechanisms, such as voltage-, ligand-, and membrane tension-dependent gating [6], these channels share similar pore domains, and hence the molecular mechanisms of ion conduction through the pore have been a long-standing conundrum. For instance, it is still unclear how the ion concentration and membrane voltage drive ion conduction [7] at the atomic level.

Continuous innovations in X-ray crystallographic techniques over the past few decades have provided fruitful information about the three-dimensional (3D) structures of potassium channels [8–12]. The first X-ray structure, reported by McKinnon and coworkers, revealed that the conduction pore is composed of a wide cavity on the intracellular side of the pore, and a bottleneck-shaped narrow pore on the extracellular side, constructed by the well-conserved signature sequence (TGVYG) called the “selectivity filter” (SF). The SF is considered to be the keystone of the ion selectivity and the ion flux control. The backbone oxygen atoms of the SF forms a series of octahedral sites allowing selective diffusion of K^+^ ions in a single-file manner [13].

Various models for single-file ion conduction have been proposed to describe the microscopic behavior of K^+^ ions in the SF. The “knock-on model” proposed by Hodgkin and Keynes states that the ion permeation proceeds by the three tightly coupled processes; *viz*. the association, the dissociation, and the movements of the ions in the SF [13]. In this model, a critical step is when the outermost ion in the SF is pushed out by an incoming ion. Recently, Nelson studied conduction models from a kinetic perspective [14,15]. According to the “Association/Dissociation (A/D) model”, proposed by Nelson, the ion permeation is a two-step process in which the association step of an ion to the SF is followed by an independent dissociation step of the outermost ion to the extracellular fluid. Nelson’s kinetic formulation argued that the A/D model more reasonably describes the ion conductance as a function of the ion concentration and the membrane voltage, as compared to the knock-on model. However, both conduction mechanisms are still controversial, because it is quite difficult to investigate the dynamic behaviors of molecular systems at an atomic level by experimental measurements.

A promising approach for solving such problems is the molecular dynamics (MD) simulation method, because it can simulate the time evolution of each atom in a molecular system, based on Newtonian mechanics. Since the first crystal structure of a potassium channel was solved by Doyle et al. [8], the MD method has been extensively applied for ion channel analyses. One major strategy is to calculate the free energy landscape of the ion conduction processes to elucidate the ion conduction paths and the ion selectivity, by using an umbrella sampling technique [3,16–22]. Bernèche and Roux presented the energy landscape of the ion conduction process of a KcsA channel, and their results showed that the knock-on model is energetically more favorable than the vacancy-diffusion model [17].

In contrast to the umbrella sampling method, which biases the motion of ions along the pore, the canonical MD method can directly simulate the time courses of the ion conduction processes in the canonical ensemble, without biases along a reaction coordinate. It allows observations of the trajectories of proteins, waters, and ions at atomic resolution. However, for ion permeation, this approach is computationally demanding because ion conduction is a rare event (~10^8^ K^+^ ions permeate through a potassium channel in one second under physiological conditions), and thus long-term simulations are required to sample repeated ion conduction events. Early trials to simulate the time courses of ion conduction in K^+^ channels successfully observed a small number of ion conductions within several tens of nano-seconds of simulation times [23,24], while recent advances in computer technologies have made it possible to perform long-term simulations. Notably, Jensen *et al*. successfully simulated micro-seconds of the ion conduction processes of Kv1.2 [25,26], by using specialized hardware for the MD simulations [27]. In their studies, they concluded that the ion conduction processes occurred via the knock-on model. However, it should be noted that their simulation results revealed a sub-stable, intermediate state between the association and dissociation of ions, and Nelson argued that it is a variant of the A/D model; namely, the asymmetric “shunt-on pop-off” model [15].

Previously, we performed micro-second scale MD simulations of the pore domain of a Kv1.2/2.1 chimeric channel under several different ion concentrations. We found that there are two major mechanisms, and the preference between the two mechanisms strongly depends on the ion concentrations [28]. In the first mechanism, the pore in the resting state (three-ion bound state) catches another K^+^ ion from the intracellular fluid (four-ion bound state), and then the outermost ion is readily pushed out to the extracellular side to return to the resting state. In this report, we would like to call this mechanism “III-IV-III mechanism”, in terms of the trajectory of the number of ions in the pore. In the alternative mechanism, the outermost ion of the resting state is released before the incoming ion is captured from the intracellular side (two-ion bound state), which we call the “III-II-III mechanism”. Note that we refer to these mechanisms as knock-on and A/D, respectively, in terms of the coupling of the association and dissociation of ions, but the terminology of the conduction mechanisms is ambiguous and depends on the context. Therefore, in this report, we refer to the two models as the III-IV-III and III-II-III mechanisms, respectively.

In this report, we studied the ion conduction mechanisms of the pore domain of the potassium channel, taken from the same Kv1.2/2.1 paddle chimeric structure [10] as in the previous work, by using MD under several different electric field strengths. We analyzed the 6.5 μs simulation trajectories in total by integrating them with the trajectories generated in our previous study (the 3.0 μs data were from the previous work, and the 3.5 μs data were generated in this work). The fact that both conditional changes; *i*.*e*., increasing the ion concentrations and the voltage, accelerated the ion current would naively suggest that the two conditions have similar effects on the conduction mechanisms. However, our simulations showed the opposite result. Increases in the ion concentration and the voltage drive ion conduction by different mechanisms. These results indicate that the preference of the microscopic ion conduction mechanism is determined by a balance between the effects from the ion concentration and the electric field.

## Materials and Methods

### Molecular Dynamics Simulations

**Fig 1:**
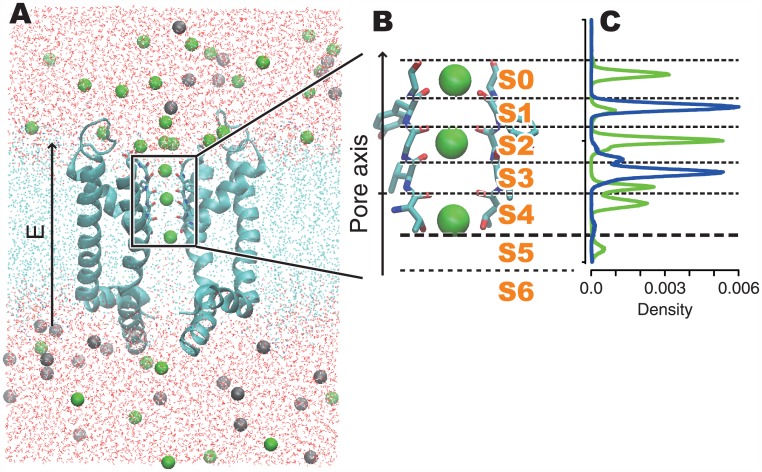
The simulation system and the definitions of the ion-binding sites in the pore. (A) The entire structure of our simulation system. The cyan ribbons represent the pore domain of homo-tetrameric Kv1.2 (part of the structure PDB-ID: 2r9r), and only two of the four subunits are shown for clarity. Green and gray balls are K^+^ and Cl^−^ ions, respectively. Cyan dots are atoms composing the POPE lipid bilayer. The oxygen and hydrogen atoms of the water molecules are shown as red and white dots, respectively. In the figure, the bottom and top correspond to the intra- and extra-cellular sides across the membrane, respectively. During the simulations, a constant electric field was applied along the direction from the intra- to extracellular side. (B) The structure of the SF and the definition of the ion-binding sites. The sticks indicate the structure of the signature sequence (TVGYG) of two of the four subunits. The ion-binding sites were defined by certain threshold values along the pore axis coordinates (see the main text). (C) Density plot of K^+^ ions (the green line) and water molecules (the blue line) under the +1,226 mV conditions.

A series of MD simulations were performed on the system consisting of the pore domain of Kv1.2 (obtained from the chimeric structure at the paddle with Kv2.1, in the rat brain; PDB-ID: 2r9r), a POPE lipid bilayer, and a 150 mM KCl solution (Fig 1A). The simulation system and the methods were the same as those used in our previous study [28], except for the strength of the constant electric field. We performed simulations under electric fields corresponding to -1,380 mV, -920 mV, -460 mV, 0 mV, +460 mV, +1,073 mV, +1,226 mV of the membrane voltages. In addition, four trajectories generated in our previous study, which are under +920 mV of the membrane voltage with 150 mM, 300 mM, 450 mM and 600 mM of KCl concentrations, were also analyzed. Although the values of the voltage and ion concentrations are higher than those in the physiological conditions, it was necessary to observe a considerable number of K^+^ ion permeation events This approach was employed to overcome the difficulty of the underestimation of K^+^ currents reported by many MD studies [25, 26, 28, 29]. Such high voltages and ion conditions are also effective in emphasizing the effects of these conditions. The membrane voltage *V* was calculated as *V = L*_*Z*_*E*, where *E* denotes the strength of the electric field, and *L*_*Z*_ denotes the length of the simulation cell in the z-axis [30], where the membrane is placed parallel to the xy-plane. Note that the definition of *L*_*Z*_ in the paper published in 2010 by Jensen *et al*. [25] was the length of the SF, and that in Bernèche’s paper published in 2000 [23] was the thickness of the membrane. This issue was fully investigated by Roux’s group [30,31]. For all seven conditions, 0.5 μs simulations were performed. In total, a 6.5 μs trajectory was analyzed, including 3.0 μs from the previous study. In each run, the trajectory in the first 0.05 μs was considered as an equilibration process and was eliminated from the analyses.

The simulation model was built by the System Builder module of the Maestro software [32]. The system was relaxed by applying an energy minimization, a relaxation run with gradual release of position restraints with a gradual increase of the temperature, by using the Desmond software [33]. The production runs of the MD simulations were performed by using the GROMACS software [34] with the NPT ensemble, allowing semi-isotropic expansion of the periodic cell at 300 K and 1.0 bar, based on the Parrinello-Rahman barostat [35,36]. The standard Charmm27 force field [37] with CMAP correction [38] was applied for potential energy calculations of peptide chains, lipids, and ions. Water molecules were modeled with the TIP3P model. The electrostatic energies were calculated by using the particle mesh Ewald method [39]. The covalent bonds with hydrogen atoms were constrained by the LINCS algorithm [40].

### Ion-binding State Analysis

The conduction mechanisms observed in the simulation trajectories were analyzed by using the “ion-binding state analysis” method, proposed in our previous report [28]. First, we defined the pore axis, as the vector from the center of the Thr370 carbonyl oxygen atom in each subunit to the center of that of Tyr373. The trajectories of the K^+^ ions were analyzed as one-dimensional motions along this pore axis. Next, seven ion binding sites, from *S0* to *S6*, were defined by threshold values in the pore axis at the positions of the minimum potassium existing densities (Fig 1B and 1C). Here we used the same thresholds as those defined in our previous report, based on the simulation at a +920 mV voltage and a 150 mM ion concentration. Note that, the names of ion binding sites have been referred to as S0 through S4, and the remaining hydrated region of the pore has been referred to as the cavity. In this report, we divided the cavity into S5 and S6, that are the partially hydrated ion-binding site formed by the side-chains of Thr370, and the fully hydrated cavity. Then, the ion-binding state of each snapshot was presented in terms of which ion-binding sites retain K^+^ ions. For example, when K^+^ ions are bound at *S0*, *S2*, and *S4*, the ion-binding state is referred to as *K*:*0*:*2*:*4*. The trajectory of the simulation can be transformed into a series of ion-binding states; *e*.*g*., “*K*:*0*:*2*:*4* at 1.0 ps, *K*:*0*:*2*:*3* at 2.0 ps, …”. The mechanisms of ion conductions will be discussed based on the time courses and the statistics of the ion-binding states.

## Results

### Current–voltage Relationship

Except for the conditions at 0 mV and -460 mV of the membrane voltage, ion conduction events were repeatedly observed during the simulations. In total, 19 and 3 K^+^ ions were permeated along the inward direction at -1,380 and -920 mV, respectively, and 8, 33, 34, and 32 K^+^ ions were permeated along the outward direction at +460, +920, +1,073, and +1,226 mV, respectively. These values correspond to currents (and their bootstrap standard errors) of -6.1 (1.4) pA, -1 (0.55) pA, 2 (0.72) pA, 5.3 (0.62) pA, 11 (1.9) pA, and 10 (1.8) pA for each voltage in the ascending order. The bootstrapping was performed, by using the local block bootstrap method [41] with 100 ps block size and 10^6^ iterations. As in Jensen’s study [26], our MD simulations underestimated the ion currents, especially in the low voltage regime. This underestimation may be due to the insufficiencies of the force field to reproduce accurate interactions of the selectivity filter and ions, or due to the other factors that were not treated in this study, *e*.*g*., complex mixture of membrane components in the physiological conditions. Here we focus on only the relative differences of the molecular mechanisms between the conditions with stronger and weaker electric fields, and avoid discussing the quantitative correctness of the ion current.

We confirmed that the systems were stable during the long simulations even under the extremely high voltages. The RMSD values of the backbone atoms under +1,226 mV voltage were less than 1.6 Å during 0.5 μs simulation and the SF kept the low-K^+^ conformation [42], however some reversible conformational changes of the SF were observed (S1 Fig). A dihedral motion at Gly376 slightly collapsed the octahedral ion-binding sites S2. This conformational change was observed twice in the trajectory at 250~320 ns and 400~430 ns, but they readily recovered the native conformation. Ions could not pass through S2 when the site was disrupted (S1 Movie). On the other hand, the surface area of the membrane, which means the area of the XY-plane in the periodic boundary cell, was slightly shrunk. The difference of the area between the first and last 10 ns were only approx. 5% (S2 Fig). These results suggest that that our simulations did not cause any significant disruption of the systems.

### Ion-binding State Analysis

**Fig 2:**
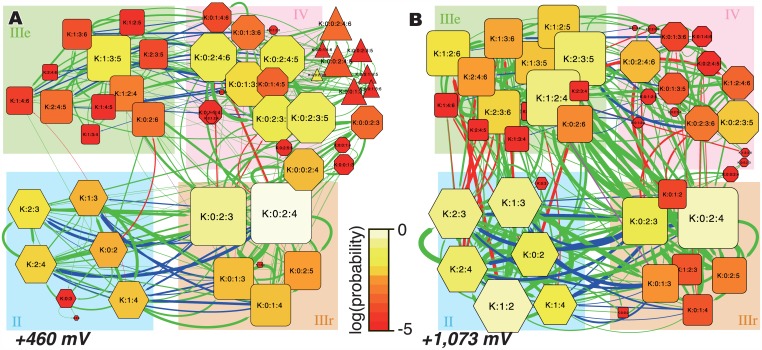
The ion-binding state graphs at (A) +460 mV and (B) + 1,073 mV. The nodes indicate the ion-binding states of the pore, and the edges indicate the transitions between them. These figures summarize the ion-binding states observed during the simulations.

In our previous study, a series of ion-binding sites in the pore, *S0–S6*, were defined from the density distribution of K^+^ along the pore axis coordinates (Fig 1C). These discretized states are useful to capture the characteristics of the complex simulation trajectories. An energy landscape of ion-binding states in a simulation trajectory can be summarized as a network representation. The networks of ion-binding states and their transitions observed during the simulations at +460 mV and +1,073 mV are shown in Fig 2A and 2B, respectively (results obtained with other voltages are shown in S3 Fig). These figures are referred as “ion-binding state graphs” in this report.

In these graphs, the nodes denote the ion-binding states of the ion channel pore, and identify which ion binding sites, *S0–S6*, retain K^+^ ions. The sizes and colors of the nodes indicate the stability of each state: brighter and larger nodes are more stable during the simulation. The shapes of the nodes reflect the number of K^+^ ions bound in the pore: hexagon, rounded square, octagon, and triangle represent two-, three-, four-, and five-ion states, respectively. The edges indicate transitions between two states. The red edges indicate transitions with a new incoming K^+^ ion from the intracellular fluid to *S6*, and the blue edges indicate those with a K^+^ ion detaching from *S0* of the pore. Therefore, an ion conduction event can be represented as a cyclic path including red and blue edges in this graph.

For all voltages, the *K*:*0*:*2*:*4* state is the most stable. We classify the most stable state and its peripheral states into the group *IIIr* in Fig 2 (the bottom right part of the graphs with the orange background); “*IIIr*” means the “resting” state with “three” ions. In addition, there are three other major groups of states. Group *II* consists of states with two K^+^ ions, group *IV* consists of states with four K^+^ ions, and group *IIIe* is the precursor state to *IIIr* (“*e*” means “entrance” to the resting state). While some five-ion states were observed during the simulations (group *V*), they are relatively rare (the triangle nodes in Fig 2).

From the resting states, one of the two major kinds of conduction events can occur. (i) A new ion attaches to *S6* from the intracellular fluid along the red edges (the state transits from *IIIr* to *IV*). Then, a K^+^ ion at *S0* is pushed out along the blue edges (from *IV* to *IIIe*), and the system returns to the resting state (from *IIIe* to *IIIr*). Alternatively, (ii) the K^+^ ion at *S0* detaches toward the extracellular side along the blue edges (from *IIIr* to *II*). Then, a K^+^ ion comes in from the intracellular side along the red edges (from *II* to *IIIe*), and the system returns to the resting state (from *IIIe* to *IIIr*). In this report, we refer to these mechanisms as (i) the III-IV-III mechanism and (ii) the III-II-III mechanism. Examples of the ion conduction events are shown in S2 and S3 Movies, for III-IV-III and III-II-III mechanisms, respectively. From another point of view, we can also categorize the ion conduction mechanisms based on the number of energy barriers. In the “one-step ion conduction”, the association and dissociation of the ions are tightly coupled, which means that the ion conduction event occurs by overcoming only one barrier. In contrast, the “two-step ion conduction” event has a sub-stable intermediate state between the association and dissociation of ions, and therefore this mechanism includes two energy barriers. They correspond to the knock-on and A/D mechanisms, respectively, as defined in Nelson’s study [15]. As consistent with our previous report [28], the III-IV-III conduction occurs via the one-step mechanism and the III-II-III conduction proceeds by the two-step mechanism, because the four-ion states are very unstable and the two-ion states are sub-stable states in our simulation conditions. The distribution of the lifetime of intermediate states, which means *II* states in the III-II-III conductions and *IV* states in the III-IV-III conductions, in each conduction events (S4 Fig) showed an energy barrier in III-II-III conductions (the average lifetimes were 9.43 ns and 2.15 ns, for III-II-III and III-IV-III conductions, respectively). These two types of ion conduction mechanisms showed kinetically distinct behavior.

### Voltage Dependence of the Ion Conduction Mechanisms

The landscapes of the ion-binding states between +460 mV and +1,073 mV (Fig 2A and 2B) are clearly different. With an increase in the voltage (from 460 mV to 1,073 mV), the two-ion states (indicated as *II*) are destabilized and the four-ion states (*IV*) are stabilized. The differences in the potential of mean forces of the four-ion states against the two-ion states under the +460 mV and +1,073 mV conditions are *ΔG*_*(II*, *IV*, *+460 mV)*_ = -2.75 kJ/mol and *ΔG*_*(II*, *IV*, *+1073 mV)*_ = 9.72 kJ/mol, respectively. As mentioned above, there are two main paths for ion conduction, the III-II-III conduction (the state transits in the order of *IIIr*, *II*, *IIIe*, and *IIIr*) and the III-IV-III conduction (*IIIr*, *IV*, *IIIe*, and *IIIr*). The voltage-dependence of the stabilities of the *II* and *IV* states suggests that the preference of the ion conduction mechanism depends on the voltage. Namely, lower voltages prefer the III-IV-III conduction rather than the III-II-III conduction, and *vice versa*. In addition to the stabilities of the ion-binding states, transitions from *II* to *IIIe* were rarely observed at +460 mV (there are only a few edges from *II* to *IIIe* in Fig 2A), in contrast to the case at +1,073 mV. Even when an *S0* ion popped out to the extracellular fluid, the back-running of a K^+^ ion from the extracellular fluid (from *II* to *IIIr*) readily occurred, rather than the influx of a new ion from the intracellular side (from *II* to *IIIe*). Thus, the three-ion conductions rarely occurred at +460 mV.

**Fig 3:**
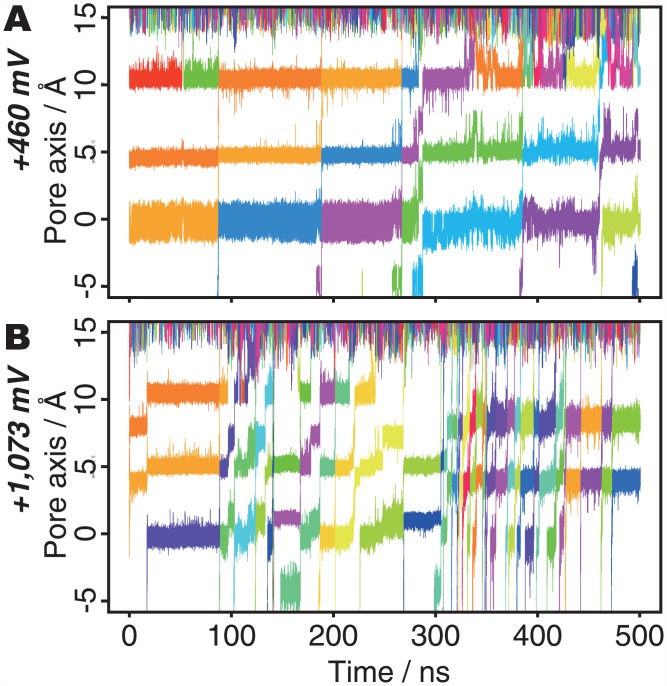
Motions of K^+^ ions in the pore axis at (A) +460 mV and (B) +1,073 mV. The colors of the plots indicate the identity of each K^+^ ion.

The pore occasionally retained five K^+^ ions at +460 mV (the triangle nodes in Fig 2A). They have two ions at *S0*, by supplying a K^+^ ion from the extracellular fluid to the pore with four ions. Many K^+^ ions accumulate around the pore exit, perhaps due to the presence of some negatively charged residues; *e*.*g*., Asp375 (S5 Fig). At lower voltages, the pore tends to retain larger numbers of K^+^ ions, and this means that the electric field provides the driving force toward overcoming the energy barrier to release the K^+^ ions from the outermost site (*S0*). The trajectory of the K^+^ ions under the low voltage (+460 mV) conditions shows that *S0* is almost always occupied (Fig 3A). In contrast, under the higher voltage conditions (+1,073 mV; Fig 3B), *S0* is vacant (or bound with water molecules) during more than half of the simulation time, and the pore frequently assumes the two-ion state. The average numbers of K^+^ ions in the pore are 3.05 and 2.49 at +460 mV and +1,073 mV, respectively. These results indicate that the electric field affects which type of ion conduction is preferred: III-IV-III and III-II-III conductions are facilitated at low and high voltages, respectively.

In addition to visualizations of the energy landscape, the conduction mechanism of each conduction event was scrutinized as a cyclic path on the graph, and the numbers of the III-IV-III and III-II-III conductions were counted at each voltage. The ratios of the III-IV-III conductions were 83.3% (5 III-IV-III conductions over 6 conductions), 36.4% (12 over 33), 14.7% (5 over 34), and 28.1% (9 over 32) for the ascending order of the voltage.

### Inward Currents

**Fig 4:**
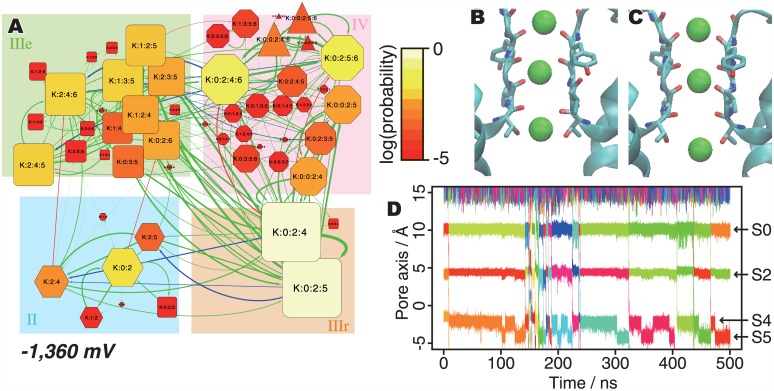
Ion conduction processes under negative (-1,380 mV) voltage. (A) Ion-binding state graph. (B and C) Snapshots of the K:0:2:4 and K:0:2:5 states, respectively. (D) The trajectory of K^+^ ions in the pore axis.

Under negative voltage conditions, inward K^+^ ion conductions were observed. The ion-binding state graph of the simulation at -1,360 mV is shown in Fig 4A. Consistent with the cases under the outward voltage conditions, stronger electric fields accelerate ion conductions and the most stable state is *K*:*0*:*2*:*4*. However, III-IV-III conductions are favored even under such strong electric fields, in contrast to the outward cases. Even with such a strong inward voltage, the *IV* states are much more stable than the *II* states (*ΔG*_*(II*, *IV*, *-1360 mV)*_ = -3.80 kJ/mol), and the majority of ion permeation events (15/19) occur along the III-IV-III mechanism. An example of this process can be visualized in S4 Movie.

In this way, first, the system at the resting state (*K*:*0*:*2*:*4*) transits to *K*:*0*:*2*:*5* (Fig 4B and 4C). This *K*:*0*:*2*:*5* state is a sub-stable state, unlike the case of the outward voltage conditions with the sub-stable *K*:*0*:*2*:*3* state. The trajectory of the K^+^ ions along the pore axis also revealed a temporary stop of K^+^ ions at *S5* (this site is formed by the side chain of Thr370, and half of the K^+^ ion in this site is solvated) in several ion conduction events (Fig 4D). Then, the three ions proceed in the pore toward the intracellular side *(K*:*2*:*4*:*6*). Subsequently, a new ion enters from the extracellular fluid (*K*:*0*:*2*:*4*:*6*), and finally the innermost K^+^ ion in the cavity (*S6*) exits toward the intracellular fluid. During this process, several K^+^ ions accumulated around the pore entrance (before *S0*) by electrostatic interactions with Asp375, and they push ions in the pore. Eventually, the K^+^ ion at *S4* is detached and enters the intracellular fluid without the attachment of a new K^+^ ion to *S0* (the III-II-III conduction, S5 Movie).

## Discussion

**Fig 5:**
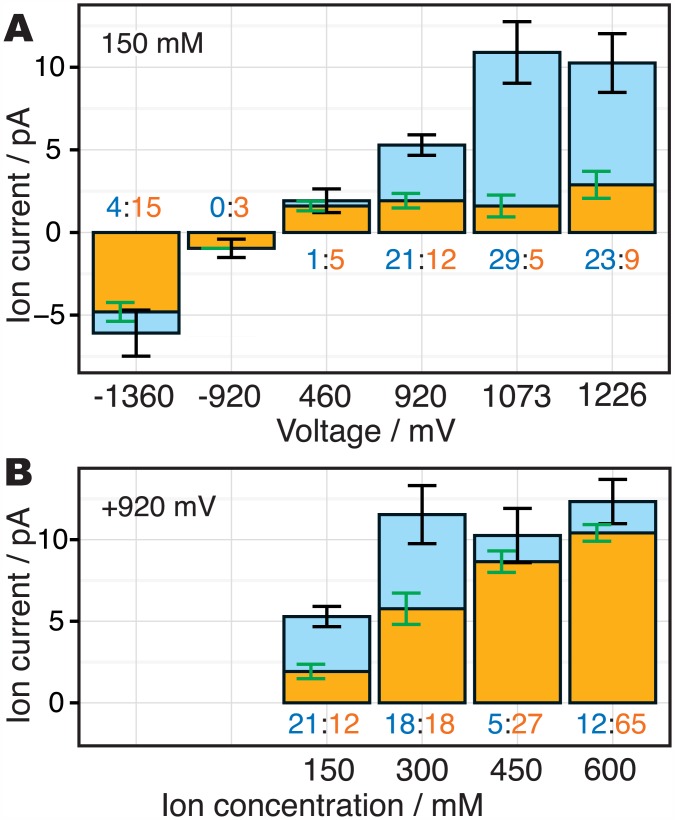
The ratios of the ion conduction mechanisms observed during the simulations. The orange and cyan bars indicate the ratios of the III-IV-III and III-II-III conductions, respectively. The orange and cyan values along with each bar indicate observed number of ion conduction events in each mechanism (note that the length of the simulation trajectories are not the same among the trajectories; the two trajectories, 150 mM with +920 mV and 600 mM with +920 mV, are 1.0 μs and others are 0.5 μs). The error bars show the bootstrap errors of the ion current (black) and the ratio of the mechanisms (green). (A) The dependency of the ion conduction mechanisms on the membrane voltage. (B) The dependency of the ion conduction mechanisms on the ion concentrations, taken from our previous study [28].

We previously reported the dependency of the ion conduction mechanisms on the ion concentration, and concluded that high ion concentrations tend to facilitate the III-IV-III conduction, due to an increase in the frequency of ions approaching the pore entrance [28]. Fig 5 summarizes the ion currents and the ratios of the III-IV-III and III-II-III conductions observed in the simulations with 150 mM ion concentrations at several voltages (Fig 5A) and those at +920 mV with several ion concentrations (Fig 5B). Interestingly, although increasing either the membrane voltage or the ion concentration raises the ion current, their effects on the conduction mechanism are contrary. These opposite effects can be interpreted from the balance of two elementary steps: attaching a K^+^ ion from the intracellular fluid to the pore, and detaching the outermost K^+^ ion from the SF to the extracellular fluid. When the attachment proceeds faster than the detachment, the pore tends to accumulate ions and the III-IV-III mechanism is preferred over the III-II-III mechanism. As an increase in the ion concentration raises the frequency of intracellular ion approaching the pore, it facilitates the III-IV-III conduction with the acceleration of the ion current. In contrast, because the membrane voltage drives the movements of dehydrated ions in the SF toward the extracellular side, it facilitates the III-II-III conduction with the acceleration of the ion current.

In short, high ion concentrations work to push the ions in the SF by introducing new ions to the pore; and strong electric fields pull dehydrated ions into the pore. However, the strong inward electric field facilitates the III-IV-III conduction, in contrast to the outward cases. Since the K^+^ ion in S5 is partly hydrated (that in S6 in the cavity is fully hydrated), pulling by the electric field does not affect it as much, as compared with the dehydrated ions. Thus, pushing the bound ions by an incoming ion largely contributes to the inward ion conduction.

In our simulations, both K^+^ ions and water molecules permeated through the channel as consistent with the streaming potential experiments on K^+^ channels which show that one water molecule is transported through the channel for each K^+^ ion passage [43]. Some theoretical studies suggested the direct Coulomb knock-on mechanism, which means water molecules do not pass through the pore [21,29]. The passage of water molecules accompanying K^+^ ions through the channel can depend on various factors, such as the desolvation energetics of K^+^ ions and the osmotic pressure across the membrane, and hence a detailed consideration is beyond the scope of this study. Although the actual microscopic phenomena that occur are still controversial, our push and pull model, explaining the effects of the ion concentration and the membrane voltage on the basis of the interactions of the innermost and outermost ions with bulk water and K^+^ ions, is not paradoxical even in the case of the direct Coulomb knock-on conduction.

In addition to the analyses of ion conduction mechanisms based on the occupancy of ion binding sites, some other interesting phenomena were observed in the simulation trajectories. In the +1,226 mV trajectory, the SF reversibly changed its conformation in sub-micro seconds time scale (S1 Movie, and S1 Fig), and this inhibits ion permeations. The modal changes of ion flux were also observed in the trajectories at +1,073 mV (Fig 3B, ca. 300~430 ns; S6 Movie, and S6 Fig) and -1,360 mV (Fig 4C, ca. 150~200 ns; S7 Movie). The ion flux in these two regimes was accelerated and there were no clear dihedral angle rotation of the SF. This result implies that the ion conduction processes include several kinds of modal changes of ion flux. As the statistical analysis of such long time scale phenomena requires longer trajectories, the molecular mechanisms of such phenomena could be studied in future works.

The pore structure other than the SF can affect on the ion conductions. Recent experimental and computational analyses have pointed out that the hydrophobic cavity, flanking the SF from the intracellular side, can account for a considerable fraction of the channel’s resistance. In particular, substitution of Pro residues with Asp at the inward edge of the hydrophobic cavity has been shown to increase the concentration of K^+^ ions in the cavity and increase the channel conductance [44,45]. An increase in K^+^ concentration in the cavity would increase the association of ions to the pore from the intracellular side, thereby enhancing permeation. In our analysis, the cavity is represented as the position “S6” (ca. 14 Å height from the bottom of S5; Fig 1B) and K^+^ ions were not stably retained in S6 position during the simulations. This means that K^+^ ions smoothly attached to the SF after entering to the cavity. We confirmed that the electric potential inside the cavity of the Kv1.2/2.1 channel without Pro to Val mutation was little different from the bulk solvent in our simulation conditions, as demonstrated by Gumbart et al. [30], and therefore the contribution of the hydrophobic cavity to the total resistance was estimated to be small. While the behavior of K^+^ ions before the entering to the cavity is out of scope of our analyses, some important phenomena may be hidden in this process.

## Conclusions

In this report, we simulated in total 6.5 μs time courses of the system consisting of Kv1.2, a POPE bilayer, and a 150 mM KCl solution, with a wide range of electric field strengths, and analyzed the ion conduction mechanisms. The results of the analyses integrated with the 3.0 μs trajectory under several ion conditions [28] revealed that the preference of the ion conduction mechanisms of the Kv1.2 pore domain is determined by the balance of the membrane voltage and the ion concentrations around the channel. Under high voltage conditions, the electric field pushes ions in the SF, and therefore the outermost ion can be released into the extracellular fluid without pushing by the fourth ion. This facilitates the III-II-III conduction, rather than the III-IV-III one. Furthermore, higher ion concentrations facilitate the III-IV-III conduction, because an increase in the ion concentration raises the chances of K^+^ ions interacting with the pore, and the approaching new ion pushes the ions in the pore by the knock-on mechanism. High voltages and high ion concentrations accelerate the ion current by different mechanisms.

In the cellular environment, the local ion concentration around a channel and the membrane voltages are dynamically changing. Therefore, the preferences of the III-II-III and III-IV-III ion conduction mechanisms are also dynamically shifting in the cell during the polarization-depolarization cycle. Since these two ion conduction mechanisms should have distinct kinetic features, the differences in the conduction mechanisms can contribute to controlling the ion flux.

## Supporting Information

S1 Fig Conformational changes at +1,226 mV. A) The time course of the backbone RMSD value of the entire channel. B) The time course of the backbone RMSD of the SF, which is the residues Thr373 through Gly378. The arrows with labels “C” and “D” indicate time points corresponding to the snapshots in the panels (C) and (D), respectively. C and D) The snapshots of the SF at 235 ns (C) and 275 ns (D). Only two of the four subunits are shown for clarity. The observed conformational change around Gly376 is emphasized by the dashed pink circles.
(EPS)

S2 Fig The time courses of the area of the XY-plane, which is the perpendicular to the pore axis, at A) +920 mV and B) +1,226 mV. (EPS)

S3 Fig Ion-binding state graphs at (A) -920 mV, (B) -460 mV, (C) 0 mV, and (D) +1,226 mV. See the legend of Fig 3.
(EPS)

S4 Fig The lifetime distributions of the intermediate states in III-II-III and III-IV-III conduction events, observed under the positive voltages conditions (+460 mV, +920 mV, +1,073 mV, and +1,226 mV). The same data are presented in two ways: (A) the boxplot, depicting the first quantile, median, third quantile, and outliers, and (B) the histogram. As the lower voltage stabilize the four-ion states, the III-IV-III lifetime in +460 mV conditions tends to be long (the green bars in B). The schematic illustrations of (C) III-II-III and (D) III-IV-III mechanisms are shown. The dashed arrows indicate the range of the intermediate, which is defined by the range from just after leaving from the resting state to just before reaching to the resting state.
(EPS)

S5 Fig A snapshot of a structure with two K^+^ ions in S0. Only two of the four subunits are shown for clarity.
(EPS)

S6 Fig Ion-binding state graphs at +1,073 mV during (A) 0~300 ns and (B) 300~500 ns. The two time ranges show distinct behavior. The latter part of trajectory much prefers the states II than the first part does.
(EPS)

S1 Movie Conformational changes of the SF, observed in the simulation at +1,226 mV. This movie depicts the time course of 500 ns in the simulation time (500 ps per frame). The trajectory was smoothed in order to emphasize the conformational changes of the backbone.
(M4V)

S2 Movie An example of the III-IV-III conduction process, observed in the simulation at +460 mV. This movie depicts the time course of 20 ns in the simulation time (20 ps per frame).
(M4V)

S3 Movie An example of the III-II-III conduction process, observed in the simulation at +1,073 mV. This movie depicts the time course of 16 ns in the simulation time (20 ps per frame).
(MPG)

S4 Movie An example of the III-IV-III inward conduction process at -1,360 mV. This movie depicts the time course of 1 ns in the simulation time (1 ps per frame).
(M4V)

S5 Movie An example of the III-II-III inward conduction process at -1,360 mV. This movie depicts the time course of 1 ns in the simulation time (1 ps per frame).
(M4V)

S6 Movie A modal ion flux change observed in 250~350 ns of +1,073 mV trajectory (100 ps per frame). See also Fig 3B.
(MPG)

S7 Movie A modal ion flux change observed in 100~200 ns of -1,360 mV trajectory (100 ps per frame). See also Fig 4D.
(MPG)
